# The Impact of Limited Vaccine Access on COVID-19 Mortality—Descriptive Study of COVID-19 Vaccination and Mortality Due to COVID-19 in Montenegro, July 2020–February 2022

**DOI:** 10.3390/vaccines13030278

**Published:** 2025-03-06

**Authors:** Aleksandar Obradović, Marija Raičević, Milko Joksimović

**Affiliations:** Institute for Public Health of Montenegro, 81000 Podgorica, Montenegro

**Keywords:** COVID-19, vaccination, mortality, vaccine inequity, vaccine hesitancy

## Abstract

**Introduction**: The Delta variant of SARS-CoV-2 dominated Montenegro from July 2020 until early 2022, when Omicron took over. COVID-19 vaccination began on 20 February 2021, two months later than in the EU. The study aimed to investigate the impact of vaccination on mortality rates in Montenegro during Delta’s predominance. **Methods**: A descriptive study was conducted using data from the Montenegrin Institute of Public Health COVID-19 database, the Population Electronic Immunization Register, which provides data for all administrated COVID-19 vaccines in Montenegro, and EUROSTAT mortality data. **Results**: COVID-19 accounted for 17.8% of total deaths in Montenegro during the period of study. Crude mortality rate among unvaccinated was almost four times higher compared to those who received at least one vaccine dose. Inactivated vaccines were predominantly administered to those aged 60 and over, while RNA vaccines were mainly given to those under 60. The median interval between the last vaccine dose and death was significantly higher for vector vaccines compared to inactivated vaccines. **Discussion**: The delayed start of vaccination and limited vaccine choices in Montenegro likely contributed to prolonged high mortality due to COVID-19. Our findings reveal disparities in vaccine distribution and effectiveness, highlighting the need for timely and equitable access to effective vaccines, especially for vulnerable populations. **Conclusions**: The study underscores the importance of prompt vaccine distribution and the option to choose vaccine types in building public trust and reducing mortality rates. It emphasizes the need for strengthening global mechanisms COVAX and continuing educational activities to address vaccine hesitancy and ensure equitable access to effective vaccines.

## 1. Introduction

Since its emergence in late 2019, the COVID-19 pandemic, caused by the SARS-CoV-2 virus, has impacted global public health in many ways. As the virus continued to spread and evolve, various genetic mutations led to the emergence of different variants, each potentially possessing distinct characteristics in terms of transmissibility, virulence, and immune evasion capabilities. According to data from the Institute for Public Health of Montenegro, the delta strain of SARS-CoV-2 became predominant in Montenegro in July 2020. This coincided with the onset of increased mortality due to the COVID-19 pandemic, as recorded in the World Health Organization’s (WHO) estimation of excess mortality. The phase of elevated mortality persisted until early 2022, when a shift in the dominant strain occurred, with the Omicron variant of SARS-CoV-2 becoming prevalent in circulation [1,2,3].

Vaccination against COVID-19 commenced in Europe at the end of December 2020, with both RNA and vector vaccines simultaneously [4]. However, Western Balkans countries, except Serbia, experienced delays in receiving initial vaccine doses [5]. In Montenegro, vaccination began on 20 February 2021, facilitated by a donation of vector vaccines (a type not licensed in the European Union). Inactivated vaccines became available on 3 March 2021, while RNA vaccines were not accessible until early May 2021, when limited doses were obtained. The vector vaccine predominantly used in the European Union became available in Montenegro on 28 March 2021 [6,7,8,9,10].

The implementation of COVID-19 vaccination in Montenegro was conducted in accordance with the National Strategy for the Introduction, Distribution, and Application of COVID-19 Vaccines and the recommendations of the National Immunization Technical Advisory Group (NITAG) regarding priority groups. This strategic approach was crucial given the limited availability of vaccine doses. Mass vaccination of the general population commenced on 4 May 2021. Recommendations for administering the first booster dose were initially issued in early August 2021 for a selected group of highly vulnerable individuals. Subsequently, a general recommendation for booster vaccination was extended to the broader population at the end of September 2021 [11,12,13,14].

On the other hand, the lack of vaccine options was particularly concerning, as it may have influenced vaccination uptake and overall protection within these high-risk populations. Vulnerable groups, such as the elderly, individuals with preexisting chronic conditions, and immunocompromised individuals, rely on access to vaccines that are not only effective but also have safety profiles tailored to their specific health needs.

The study aimed to investigate the impact of COVID-19 vaccination timing and type on mortality rates during the Delta variant’s prevalence in Montenegro. It examined the relationship between vaccine distribution and death rates, focusing on how the timing of vaccine allocation to high-risk groups might have affected overall mortality.

## 2. Materials and Methods

We conducted a descriptive study using multiple data sources:

(a) The Institute of Public Health of Montenegro’s COVID-19 database provided records of reported COVID-19 deaths from July 2020 to February 2022, including the vaccination status of deceased individuals.
(b) All-cause mortality data for Montenegro for the relevant period were extracted from the publicly accessible EUROSTAT database.
(c) COVID-19 vaccination coverage data for the general population of Montenegro from February 2021 to February 2022 were obtained from the Population Electronic Immunization Register maintained by the Institute of Public Health of Montenegro [15].

Data visualization was performed using Microsoft Excel (Office 365 suite), while statistical analyses were conducted using the EZR (Easy R) plugin (version 1.68) on R Commander (version 2.9-1).

To estimate the protection conferred by different COVID-19 vaccine types, we evaluated the median interval between the last administered vaccine dose and COVID-19-related death. Statistical significance was assessed using a non-parametric Kruskal-Wallis test for more than two independent samples. Subsequent pairwise comparisons were performed using the Mann-Whitney U test with Holm’s correction for multiple comparisons.

For crude mortality rate calculations, denominators were derived from two primary sources: the most recent census data for Montenegro from 2023 and the number of fully vaccinated individuals as recorded in the Electronic Immunization Register.

For this study, an individual was considered vaccinated if the period between the first received dose and death due to COVID-19 exceeded 14 days. Fatalities occurring within 14 days of receiving the first vaccine dose were not classified as deaths among vaccinated individuals.

Vaccines were grouped for analysis based on their type (e.g., mRNA, viral vector, inactivated) without considering different manufacturers.

## 3. Results

From the beginning of July 2020 until February 2022, a total of 228,687 COVID-19 cases were reported to the Institute for Public Health of Montenegro.

In the same period, 2669 people died from COVID-19, of which almost two-thirds (62.6%) were men. Such mortality led to the positioning of COVID-19 as the main cause of death in 17.8% of deaths in the same period (Table 1).

On the other hand, from the beginning of the implementation of COVID-19 vaccination in Montenegro up to the end of February 2022, number of COVID-19 deaths among unvaccinated persons was almost five times higher than that observed in vaccinated individuals, regardless of the type of vaccine. (1590 deaths among unvaccinated compared to 360 deaths among those who received at least one dose of the COVID-19 vaccine) (Table 1).

**Figure 1:**
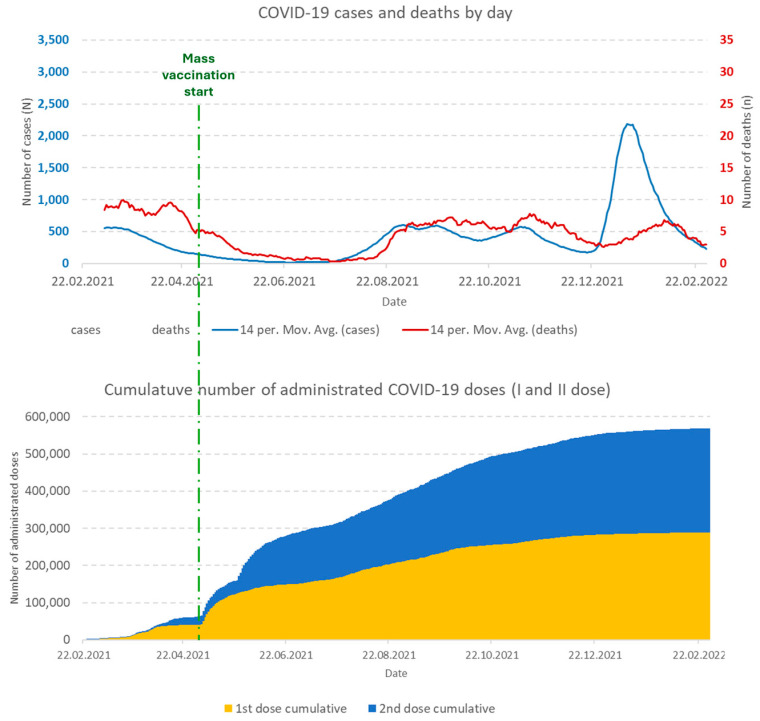
Timeline of COVID-19 cases, deaths, and vaccination (I and II doses) in Montenegro, from 20 February 2021 to 28 February 2022.

The delta strain of the SARS-CoV-2 maintained a high mortality from COVID-19 in Montenegro until the beginning of mass immunization against COVID-19 (4 May 2021). The situation worsens again, first during the summer of 2021. Then, in the period December 2021–January 2022, when the Omicron strain of SARS-CoV-2 appeared in Montenegro, the country reached the highest number of reported cases in one day (Figure 1).

During the autumn of 2021, the number of persons vaccinated against COVID-19 reached a plateau, and the total number of fully vaccinated (with 2 doses, regardless of the type of vaccine) in February 2022 was almost 280,000 persons (44.9% of Montenegrin population) (Figure 1).

**Figure 2:**
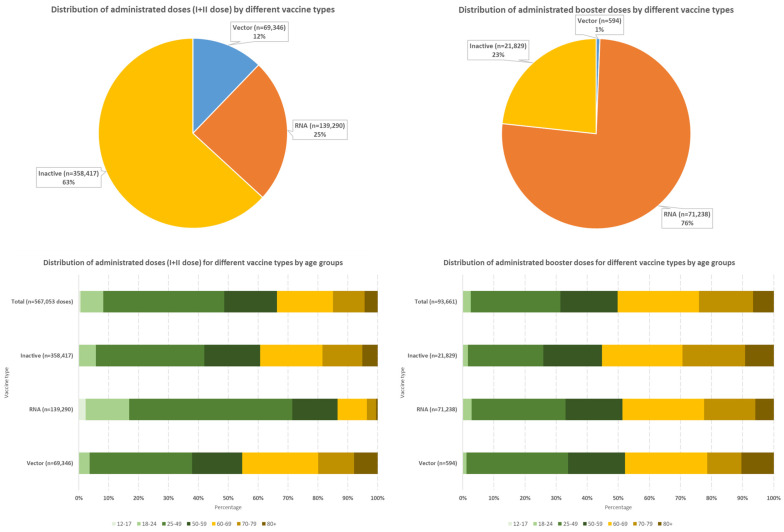
Distribution of administrated COVID-19 vaccines by different doses, vaccine types, and age groups in Montenegro, period 20 February 2021–13 February 2022.

From the beginning of the COVID-19 vaccination campaign in Montenegro up to 13 February 2022, a total of 567,053 doses of vaccine were administered as first and second doses, and an additional 93,661 doses were given as so-called booster doses (Figure 2). While the largest share among the first and second administrated vaccine doses had inactivated vaccines (slightly less than 2/3 of the total doses of the first and second doses of the vaccine), the situation was different among the booster doses, where more than 3/4 of the administered doses were RNA vaccines (Figure 2).

Almost 40% of inactivated vaccine doses received as part of primary vaccination were given to people aged 60 and over, while more than 85% of RNA vaccine doses in primary vaccination were given to the population under 60 years of age (more than 70% under 50 years of age) (Figure 2).

Regarding the vaccine type, there was no difference in the distribution of administered booster doses between the age categories younger than 60 years and 60 years and older (Figure 2).

**Figure 3:**
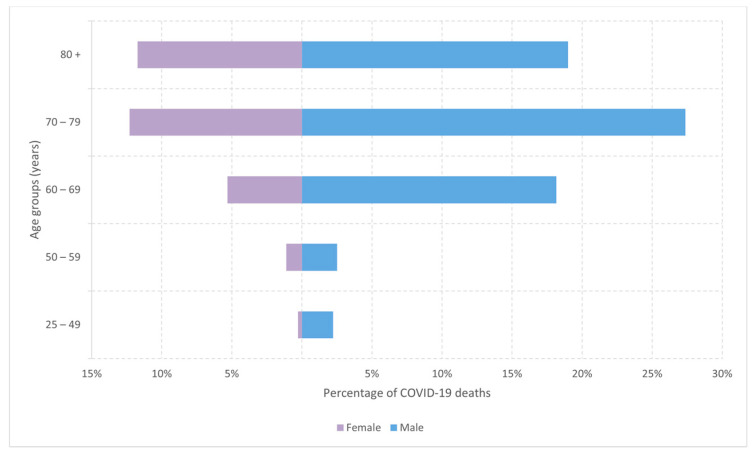
Distribution of COVID-19 deaths among COVID-19 vaccinated by gender and age groups (n = 360), period 20 February 2021–28 February 2022.

The highest number of COVID-19 deaths among those who received at least one dose of the COVID-19 vaccine was recorded among people aged 60 and over, and in all age groups, there were more men among the deceased. The gender disparity in COVID-19 deaths was most pronounced among individuals aged 60 to 69, where there were three times more COVID-19 fatalities among males (Figure 3).

Comparison of medians of interval in days between the last dose of received COVID-19 vaccine and death event by Kruskal-Wallis test (Mann-Whitney U test—Holm) showed significantly higher median among those who were fully vaccinated with vector type of vaccines compared to those who were fully vaccinated with inactive type of COVID-19 vaccines (Kruskal-Wallis chi-squared = 7.7961, df = 2, *p*-value = 0.02028) (Table 2).

Also, for all three types of COVID-19 vaccine, in more than 75% of cases, this interval was at least approximately 4 months (Table 2).

## 4. Discussion

During the study period, COVID-19 emerged as the second leading cause of death in Montenegro, accounting for 17.6% of total registered deaths with a crude mortality rate of 600.8 per 100,000 inhabitants. This surpassed the estimated annual mortality attributed to malignant neoplasms, highlighting the pandemic’s profound impact on Montenegro’s mortality profile. This situation slightly differs from the broader trend observed in 2020, where COVID-19 was the third leading single underlying cause of death in many countries, including those in Europe, surpassed only by heart disease and malignant neoplasms [16,17].

Our analysis revealed a statistically significant higher median interval between the last dose administration and fatal outcome for vector vaccines compared to inactivated vaccines. However, the data suggest a time-limited protective effect for all COVID-19 vaccines administered in Montenegro, regardless of vaccine type. This temporary protection against COVID-19 fatality is evidenced by the 25th percentile of the interval between vaccination and death, which consistently approximates or exceeds 120 days across all vaccine types (Table 2). This finding indicates that while vaccine efficacy may vary, all types demonstrate a similar pattern of waning protection over time, emphasizing the potential need for timely booster doses to maintain optimal protection against severe COVID-19 outcomes.

The crude mortality rate from COVID-19 among vaccinated individuals was observed to be approximately four times lower compared to unvaccinated individuals, regardless of the administered COVID-19 vaccine type. This marked difference in mortality rates underscores the critical role of COVID-19 vaccination in mitigating fatal outcomes, providing compelling evidence for the efficacy of immunization strategies in reducing severe disease and death associated with SARS-CoV-2 infection (Table 1).

Studies have shown significant differences in the coverage of COVID-19 vaccination between countries, especially between high- and low-income countries, and Montenegro is classified as an upper-middle-income country. The main reasons for these differences were identified as an insufficient supply of vaccines, unequal distribution, insufficiently strong health systems, and consequently, a high degree of hesitancy when it comes to vaccination [18,19,20,21].

Within a year of the start of distribution, highly developed countries vaccinated up to 80% of the population, while during the same period, low-income countries vaccinated <10% of their population, largely due to inequality in vaccine access, which may be considered to represent one of the main challenges in international cooperation during the pandemic. All of this may even produce long-term consequences, including prolonged vulnerability to COVID-19 outbreaks and giving space for increased vaccine hesitancy [22]. Some examples of mathematical modeling demonstrated that the optimal vaccine allocation strategy would involve distributing vaccines based on country population size and prioritizing vaccination for high-risk groups. This approach would give significant benefits by slowing down the international spread of the disease and consequently avoiding unnecessary deaths [23].

The findings of our study align with the aforementioned research and underscore the significant consequences of delayed vaccine acquisition and distribution. Montenegro’s experience serves as a compelling case study in the broader context of global vaccine equity. Specifically, Montenegro’s procurement of initial vaccine contingents occurred approximately two months after the commencement of global vaccination efforts, with RNA vaccines not becoming available until May 2021. As a result of this lack of vaccine availability, the highest-risk groups, which were prioritized for vaccination, predominantly received inactivated vaccines. Numerous studies have consistently demonstrated that inactivated vaccines, while still beneficial, exhibit lower efficacy compared to their vector and RNA counterparts, particularly in older age cohorts. This disparity in effectiveness is especially pronounced among the elderly and those with underlying health conditions—precisely the groups most urgently in need of robust protection against COVID-19. Furthermore, research has shown that inactivated vaccines tend to confer shorter-duration immunity, requiring more frequent booster doses to maintain adequate protection levels [24,25].

Despite the initiation of mass vaccination efforts, Montenegro continued to experience high mortality rates from COVID-19, indicating that the vaccination campaign did not yield the anticipated outcomes. This situation was further exacerbated by the delayed commencement of the vaccination program. The plateau in COVID-19 vaccination coverage in Montenegro coincided with the global trend of plateauing vaccination rates but with notably lower overall coverage levels (45% of the general population vaccinated with two doses of the COVID-19 vaccine by the end of the study period) [26].

The situation in Montenegro became increasingly complex towards the end of 2021 with the emergence of the highly infectious Omicron variant of SARS-CoV-2. This development occurred approximately 4 to 5 months after COVID-19 vaccination rates had plateaued in the country, coinciding with a period when vaccine-induced immunity was beginning to wane among those vaccinated at the beginning of the vaccination campaign. The timing of the Omicron variant’s arrival was particularly unfortunate, as it exploited a window of vulnerability in the population. Stagnating vaccination rates, along with the diminishing protective effects of earlier vaccinations, create favorable circumstances for the rapid spread of this more transmissible strain. This convergence of factors—a new, highly infectious variant, plateaued vaccination rates, and waning immunity—created conditions that exacerbated the already existing public health crisis in Montenegro. All these factors certainly contributed to the complexity of the situation in Montenegro, especially when it is taken into account that the period covered by the study was a period of political turbulence in the country, in which three different state administrations were practically changed [3,27].

The inequitable distribution of COVID-19 vaccines among nations, as thoroughly documented in scientific studies, has revealed significant and concerning disparities that are strongly correlated with key socioeconomic and geopolitical factors. Specifically, research has shown that vaccine access and distribution were closely linked to a country’s GDP per capita, political stability, and ranking on the World Power Index. This uneven allocation of vaccines represents a critical failure in global health equity and cooperation.

The consequences of this inequality in vaccine distribution have been far-reaching and multifaceted, extending well beyond the immediate and devastating impact of high COVID-19 mortality rates in countries that did not receive timely and adequate quantities of efficacious vaccines. One of the most alarming repercussions has been a consequential decline in public trust towards vaccination as a whole, despite it being widely recognized as the most effective preventive measure against infectious diseases.

This erosion of confidence in vaccines has manifested in a troubling trend: a decreased uptake of almost all vaccines included in mandatory immunization schedules in the post-COVID-19 period. This phenomenon is not limited to COVID-19 vaccines but has spilled over to affect long-established vaccination programs for other diseases. The consequences of this vaccine hesitancy are already becoming apparent, with outbreaks of previously well-controlled diseases such as measles and pertussis occurring in various parts of the world.

Public health experts warn that this is likely just the beginning. There is a growing concern that it is only a matter of time before other vaccine-preventable diseases re-emerge in epidemic proportions. Diseases that were once considered under control or on the verge of eradication could potentially resurge, posing significant threats to global health security. This situation underscores the critical importance of equitable global vaccine distribution and the need for concerted efforts to restore public confidence in vaccination programs [21,28,29].

The primary limitation of this study lies in its reliance on the vaccination status of individuals deceased from COVID-19 as the principal indicator, without accounting for comorbidities and other potential confounding factors that may have influenced mortality outcomes. We did not account for comorbidities, age-specific risks, or socioeconomic factors that could influence both vaccination uptake and mortality outcomes, so this approach may oversimplify the complex interaction of variables contributing to COVID-19 fatalities. Furthermore, the study faced a significant limitation due to the delayed introduction and subsequent underrepresentation of RNA vaccines among vulnerable populations. This timing issue resulted in an insufficient sample size of deceased individuals who had received RNA vaccines. Consequently, this limitation precluded a robust comparison of the interval between the last administered dose and death events for vector versus RNA vaccines (Table 2).

## 5. Conclusions

A study on COVID-19 vaccination and mortality in Montenegro from July 2020 to February 2022 offers insights into the effects of limited and delayed vaccine access during a global pandemic. The findings highlight the critical importance of timely and equitable vaccine distribution, the need for a variety of vaccine options, and the ongoing challenge of maintaining the public’s trust in vaccination programs.

The delayed start of vaccination compared to EU countries, as well as the real lack of opportunity to choose vaccines on a country level, affected the public’s trust in them. This certainly also influenced the choice of the type of vaccine by individuals because the choice can only be made when there is an opportunity to choose, which was not the case in Montenegro until May 2021. The absence of vaccine diversity and the perception of being “left behind” in the global vaccination campaign may have contributed to vaccine skepticism and hesitancy among the population.

In addition to economic factors that highlight the need to strengthen global mechanisms, such as COVAX, it is essential to ensure the timely distribution of necessary vaccine doses regardless of the economic power of individual countries. Mass promotional vaccination campaigns and educational activities must also continue. Special emphasis should be placed on educating those who can influence attitudes about vaccination, such as decision-makers and politicians, to foster a synergistic approach to addressing this complex issue.

## Figures and Tables

**Table 1:** COVID-19 deaths in Montenegro from 1 July 2020 to 28 February 2020.

Number of COVID-19 Deaths in a Specific Time Period	Males (%)	Females (%)	Total	Median Age (IQR)	% of Total Deaths *	Crude Mortality Rate per 100,000
1 July 2020–28 February 2022	1672 (62.6)	997 (37.4)	2669	73 (65–81)	17.8	428.1
Among unvaccinated 20 February 2021–28 February 2022	790 (56.8)	600 (43.2)	1590	74 (65–81)	14.8	461.9
Among vaccinated 20 February 2021–28 February 2022	249 (55.4)	111 (44.6)	360	75 (68–81)	3.8	128.9

* % of total deaths in the same period, regardless of cause of death.

**Table 2:** Interval between the last dose of COVID-19 vaccine and death event among people fully vaccinated (n = 287 *) with different types of vaccine, Montenegro, period 20 February 2021 to 28 February 2022.

COVID-19 Vaccine Type	Interval (Days) Between the Last Dose and DeathMedian (IQR)	Pairwise Comparisons Using Mann-Whitney U test (Holm)
Inactive (n = 247)	158.0 (117.5–207.5)	Reference
Vector (n = 34)	182.5 (157.0–217.0)	*p* < 0.05
RNA (n = 6)	122.0 (118.7–150.7)	0.368

* People vaccinated with 2 doses of COVID-19 vaccine, without those who received booster dose.

## Data Availability

The raw data supporting the conclusions of this article will be made available by the authors upon request.
